# Supplementary material for: Comparative metabolism of conjugated and unconjugated pterins in Crithidia, Leishmania and African trypanosomes
Source: PLoS Negl Trop Dis. 2025 Jul 29;19(7):e0013332. doi: 10.1371/journal.pntd.0013332 (PMC12327612; doi:10.1371/journal.pntd.0013332)
Supplement: Attachment — Submitted filename: Response to reviewers.docx [file pntd.0013332.s003.docx]

PNTD-D-25-00706

Comparative metabolism of conjugated and unconjugated pterins in Crithidia, Leishmania and African trypanosomes

We thank the expert reviewers and editors for their helpful and favourable review of our manuscript. Our responses to comments are below.

**Data availability** – the figures were created in GraFit with all the data and analytical methods embedded into the supplied figures. We have extracted the relevant numerical data from the graphs (figures 2 to 5) and uploaded them as a Supporting Information Excel file.

**Competing Interests statement** – declaration completed

**Reviewers' comments:**

Reviewer #1: No issues

**Au response** – no change required

Reviewer #2: Figure 5: I have some concerns about using resazurin as an indicator of proliferation. Changes in cellular metabolic states may increase or decrease fluorescence in the assay, which does not necessarily correlate with cell proliferation. I suggest interpreting these data as cell viability rather than proliferation.

**Au response** Resazurin is widely used to determine both cell proliferation *and* cell viability in equal measure in animals, plants, fungi, bacteria and parasites (3,000 publications in Pub Med for Alamar blue or resazurin and proliferation). Reduction of resazurin to fluorescent resorufin is catalysed by an extremely wide range of metabolic enzymes in the mitochondrion and the cytosol of biological organisms and therefore minor changes in cellular metabolism (unlike cell death) are unlikely to have a marked effect on such a wide dynamic range of fluorescent signal (~1,000-fold) observed here. We are of course aware of the pitfalls of non-enzymatic reduction (e.g ascorbate, cysteine) or further reduction in cells to non-fluorescent, hydroresorufin and have included appropriate controls in our study.

However, to alert the reader to the assay method used in Figure 5, we have emphasised the growth assay is “a resazurin-based cell viability assay”.

Reviewer #3: Yes, this is a well-conducted study to explore the requirements of pterin analogous for the growth of Crithidia fasciculata, Trypanosoma brucei and Leishmania major. The methods are appropriately described and support the conclusions.

**Au response** – no change required

**Results**

**Au response** – no change required

**Conclusions**

**Au response** – no change required

**Editorial and Data Presentation Modifications?**

Reviewer #1: None

**Au response** – no change required

Reviewer #2: I believe the paper could be enriched by adding a schematic figure illustrating the metabolic pathways of pterin utilization in these parasites. This could broaden the work’s reach to other audiences.

**Au response** – a nice suggestion. We have added a new Fig 7 illustrating what is currently known on pterin metabolism.

Reviewer #3: The presentation is clear and I couldn't find possible required changes. A topic title could introduced before the ...In conclusion, (linde 388

**Au response** – We have included a sub-heading entitled “Conclusions and Future Prospects”.

**Summary and General Comments**

**Reviewer 1**

1. Since reduced pterins (BH4) is dominant intracellularly for all three parasites, I am wondering why T. brucei and Crithidia, in contrast to Leishmania, prefer oxidized pterins for growth. Does it have to do with their capacity to transport different form of pterins? Leishmania has a large family of Folate-biopterin transporters (FBT) and maybe some of them specializes in the transport of BH2 and BH4. Does T. brucei and Crithidia have an extended FBT gene family?

**Au response** – this is an interesting suggestion! We have added some speculation on this point on lines 342 to 356 in the revised version.

2. This study has not really added to our vexing lack of understanding of pterin’s function in trypanosomatids (except their growth stimulating properties). The authors hypothesized that they may have a role to play in the synthesis of unsaturated fatty acids and sterols and they provide literature of the 60s to support this hypothesis. At lines 416-418, if I understand well, the authors make a statement that they may have evidence for this. Can the authors expand on this?

**Au response** – there is nothing to add to the publication. For the interest of the expert reviewers the experimental details are as follows. C.fasciculata was cultured under low biopterin concentrations to reach stationary phase. Parasites were harvested and washed with pterin free medium by rounds of centrifugation. Cells were disrupted using a French press and fractionated into soluble and pelleted material. Cytosolic material was dialysed before use and pellets washed with pterin free medium, then sonicated to form a suspension. These extracts were then assayed for biological activity using the pterin growth assay. The next steps would have been further fractionation and bioassay were it not for the fact that my postdoc left and my lab was closed. I included this brief comment in the manuscript in the hope that someone would follow up on this very preliminary observation, which might lead to solution to this 70-year old puzzle why kinetoplastids require pterins.

3. In table 2 what are the dash (-) indicating? Not done, no inhibition?

**Au response** – no inhibition at maximum concentration tested (footnote added to table 2).

4. Title page: Why a hashtag sign for Ong?

**Au response** – Deleted (it was intended for current address, but he is no longer in research).

**Reviewer #2:** Minor revision

Lines 213–216: I did not understand the purpose of comparing PTR1 activity with trypanothione reductase activity. What insight does this provide?

**Au response** – this is used as a control to ensure complete cell disruption. Comment added at lines 213-216.

Figure 4A: The resolution is poor. Please replace it with higher-resolution images.

**Au response** – Tiff files provided for publication

Figure 5: I have some concerns about using resazurin as an indicator of proliferation.

Changes in cellular metabolic states may increase or decrease fluorescence in the assay, which does not necessarily correlate with cell proliferation. I suggest interpreting these data as cell viability rather than proliferation.

**Au response** – see comment above

Lines 404–405: I would be cautious about generalizing for all trypanosomatids based on limited examples. Including T. cruzi in the discussion might allow such generalizations for pathogenic trypanosomatids.

**Au response** – point accepted. This sentence (and elsewhere) now refers to “these trypanosomatids” since we did not include T.cruzi in this study.

I believe the paper could be enriched by adding a schematic figure illustrating the metabolic pathways of pterin utilization in these parasites. This could broaden the work’s reach to other audiences.

**Au response** – a nice, suggestion. A new Fig 7 has been added.

**Reviewer #3:** In this manuscript the authors re-explore in a comparative manner the requirements of pterin derivatives in the growth of three species of kinetoplastids. The study serves a premisse to further understand the role of these cofactors, essential in these organismos. However, the study advance quite little in the understanding of their functions.

**Au response** – no changes requested.
